# The Effect of Acute and Chronic Thermotherapy on Type 2 Diabetic Skeletal Muscle Gene Expression and Inflammatory Markers

**DOI:** 10.3390/biomedicines9091276

**Published:** 2021-09-21

**Authors:** Louay Bachnak, Jean Sparks, Daniel E. Newmire, Xavier F. Gonzales, Felix O. Omoruyi

**Affiliations:** 1 Department of Life Sciences, Texas A&M University-Corpus Christi, 6300 Ocean Drive, Corpus Christi, TX 78412, USA; jean.sparks@tamucc.edu (J.S.); Xavier.Gonzales@tamucc.edu (X.F.G.); felix.omoruyi@tamucc.edu (F.O.O.); 2 Department of Kinesiology, Texas A&M University-Corpus Christi, 6300 Ocean Drive, Corpus Christi, TX 78412, USA; Daniel.Newmire@tamucc.edu

**Keywords:** thermotherapy, type 2 diabetes, diabetic skeletal muscle cells, heat treatment type II diabetes

## Abstract

Background: Type 2 diabetes (T2D) is a chronic illness associated with resistance to or defective insulin secretion. This study investigates the effects of thermotherapy on cell viability, gene expression and inflammation in skeletal muscle cell lines. Methods: Healthy and T2D human skeletal muscle cell lines (HSMM and D-HSMM, respectively) were subjected to acute or chronic thermo-therapy (AT or CT, respectively). CT consisted of a 30 min exposure to 40 °C, three times a week for three weeks; AT was a one-time exposure. Results: A significant decrease in D-HSMM cell viability percentage followed AT; however, no significant change occurred in CT. HSMM yielded the highest elevations of genes following CT. In D-HSMM, both treatments yielded gene upregulation. Both treatments significantly down-regulated IL-1β, IL-6, IL-10 and TNF-α in HSMM. AT significantly decreased IL-1β, IL-6 and upregulated IL-10 and TNF-α levels in D-HSMM, while CT yielded a reduction in IL-4, TNF-α and an upregulation of IL-6 and IL-10. Conclusions: An increase in gene expression indicates actin activity and cellular responses, suggesting an increase in transcriptional regulation. The upregulation of IL-6 and IL-10 in D-HSMM negatively correlated with a decrease in TNF-α and IL-1β, indicating improved adverse inflammatory effects associated with the disease.

## 1. Introduction

Diabetes mellitus is a chronic metabolic disease affecting millions of people globally. It is classified as a heterogeneous disease where blood glucose levels are elevated above normal, a condition commonly known as hyperglycemia [1,2]. The disease is classified into four general groups, type 1 diabetes, type 2 diabetes (T2D) and other specific types of diabetes due to other causes that include neonatal diabetes and maturity-onset diabetes of the young, exocrine pancreas diabetes (cystic fibrosis and pancreatitis) and drug- or chemical-induced diabetes, as in glucocorticoid use in the treatment of HIV/AIDS or after organ transplantation, and gestational diabetes. The most common form of diabetes is T2D, accounting for 90–95% of all diabetes [1,3]. Previous studies have shown low muscle mass and strength are associated with an increased risk of T2D [4].

In older populations, T2D also appears to exacerbate the progression of sarcopenia, or low muscle mass, which likely results in a greater promotion of hyperglycemia [4]. Additionally, age-related declines in muscle quality, including increased mitochondrial dysfunction and fat infiltration, are also implicated in skeletal muscle inflammation and subsequent insulin resistance [4]. Skeletal muscle is the largest insulin-sensitive tissue in the body; it represents about 40–50% of total body mass and accounts for 80% of glucose uptake under euglycemic hyperinsulinemic conditions [4,5]. As the largest insulin-sensitive tissue [6], skeletal muscles play a vital role in healthy aging and muscle mass loss is present in various diseases, including sarcopenia, cancer cachexia and bed rest, displaying the importance of growth and maintenance of muscle mass [5].

Passive heat therapy is an emerging intervention to improve various health-related illnesses, including cardiovascular health [7] and skeletal muscle metabolism. This viable alternative is potentially beneficial for patients with limited exercise capabilities and includes the elderly, obese and diabetics. Humans show an adaptation to thermoregulation and the capacity to work in hot environments when exposed to chronic heat [8].

A recent study assessed the role of hyperthermia on skeletal muscle cells and affirmed physiological hyperthermia increases the mRNA responses to receptor-mediated IL-6 gene expression in skeletal muscles [9]. Previous studies also showed an increase in the body’s core temperature hindered lipopolysaccharide-induced IL-6 secretion in inflammatory cells and fibroblasts [10]. In gastrointestinal enterocytes, hyperthermia amplifies IL-1β-induced IL-6 [9]. In a letter to the Editor, Hooper et al. assessed the beneficial effects of stimulating exercise using a hot tub with T2D patients [11]. After the three weeks of exposure, the study reported a decrease in the patients’ weight, fasting plasma glucose levels and an increased sense of well-being. To probe into the effect of passive heat therapy on the skeletal muscle of diabetics, we investigated the effect of acute and chronic heat treatment on cell viability, gene expression and inflammatory markers in T2D and healthy human skeletal muscle cells. We hypothesize that cell count and anti-inflammatory markers increase following a chronic heat exposure in D-HSMM. We also hypothesize an increase in pro-inflammatory marker expression following the acute treatment in both HSMM and D-HSMM.

## 2. Materials and Methods

### 2.1. Skeletal Muscle Cell-Line Demographics

Two deidentified skeletal muscle cell lines were purchased from Lonza Inc., Walkersville, MD—healthy human skeletal muscle myoblasts (HSMM) and T2D human skeletal muscle myoblasts (D-HSMM). According to Lonza Inc., the cells were isolated from donated human tissue following informed and legal consent. The HSMM cell line was acquired from a 38-year-old Caucasian male with a BMI of 26, while the D-HSMM cell line was acquired from a 68-year-old Caucasian male with a BMI of 33.4. Upon further communication with Lonza, additional donor characteristics were unavailable. Additionally, it is worth noting the inherent variability in T2D phenotypes is due to genetic, lifestyle and environmental variation [1]. Due to this variation, the metabolic responses may be more relative to the donor and less generalizable. Our overarching goal is to explore the impact of thermotherapy on T2D skeletal muscle cell metabolism in a more controlled environment that may lead to further questions that will require more controlled study designs.

### 2.2. Media Preparation

Skeletal Muscle Growth Media-2 (SKGM-2) was prepared by mixing contents of the SkGM-2 SingleQuots Kit (Lonza Inc., Walkersville, MD, USA) into SkBM-2 Basal Medium (Lonza Inc.), followed by 5 mL of Penicillin-Streptomycin (1000 units/mL) (Thermo Fisher Scientific, Waltham, MA, USA)—referred to as SkMM. The SkGM-2 SingleQuots Kit included 0.50 mL of GA-1000, 0.50 mL of hEGF, 0.50 mL of Dexamethasone, 50.00 mL of FBS and 10.00 mL of L-Glutamine. The filtration of the mixture was performed in a sterile environment inside a biosafety cabinet while being aliquoted into 50 mL centrifuge tubes. The tubes were stored at −20 °C; the medium was thawed and warmed to 37 °C before usage.

### 2.3. Skeletal Muscle Cell Maintenance

For the initial seeding of each cell line, 5 mL of SkMM was added into a 25 cm^2^ culture vessel and incubated for 45 min at 37 °C and 5% CO_2_. Under a biosafety cabinet, the caps of the cryovials were briefly twisted to relieve pressure and then retightened. Both HSMM and D-HSMM cryovials were placed in a 37 °C water bath for a maximum of 2 min. In a biosafety cabinet, the cells were resuspended using a micropipette and the cryovial content was transferred into the appropriate 25 cm^2^ culture vessel. The vessels were rocked back and forth to evenly distribute the content, labeled and stored in a 37 °C and 5% CO_2_ incubator (VWR, Radnor, PA, USA). Upon reaching 70% confluency, the T-25 culture vessels were transferred into a T-75 culture vessel for increased surface area.

SkMM was changed every other day, or when the its color began to change. In a sterile environment, the original media were aspirated from the culture vessels and the same amount of fresh warmed medium was added. The culture vessel was then stored in the 37 °C and 5% CO_2_ incubator.

### 2.4. Sub-Culturing the Culture Vessel

Cell lines were passed when the confluency of the vessels reached 70%. The growth medium was aspirated from the culture vessel and the cells were rinsed with 5 mL of room temperature HEPES (4-(2-hydroxyethyl)-1-piperazineethanesulfonic acid) buffer saline solution (HBSS) to neutralize the complex proteins in the growth medium that may inactivate trypsin. A volume of 2 mL of trypsin was added to the vessel and incubated at 37 °C for 6 min to allow the cells to detach from the culture vessel. Under a microscope, the vessels were checked to confirm detachment at the end of the 6 min. To neutralize the trypsin, 4 mL of trypsin neutralizing solution (TNS) was then added to the 25 cm^2^ vessel.

The detached cells-and-solution mixture was transferred to a sterile 15 mL centrifuge tube. The culture vessel was examined under a microscope to ensure a successful harvest with less than 5% remaining cells. The 15 mL centrifuge tube was centrifuged at 200× *g* for 5 min to pellet the cells. The supernatant was then aspirated from the centrifuge tube, where 2 mL of growth medium was added to the pellet before the centrifuge tube was vortexed and set for cell count. 

A volume of 100 µL of the 2 mL was used for cell counting and the remaining mixture was transferred into a new sterile 75 cm^2^ culture vessel with 13 mL of growth medium and stored in the incubator at 37 °C and 5% CO_2_. 

### 2.5. Skeletal Muscle Cell Count and Viability

Cell counting was determined using a hemacytometer. In a sterile 0.5 mL Eppendorf tube, 100 µL of 0.4% Trypan Blue (Thermo Fisher Scientific) was mixed with 100 µL of cells from the 2 mL mix in the 15 mL centrifuge tube. The microcentrifuge tube was vortexed to ensure even distribution and 10 µL of the mixture was loaded into the hemacytometer underneath the coverslip. Using a microscope with a 10× objective and a tally counter, the live, unstained cells were counted. The dead, stained cells were also documented. Cell viability was calculated by (live cell count/total cell count) × 100. Total cell count was determined by adding the live cells total and the dead cells total. To determine density, cells/µL, the following equation was used: (viable cell count/5) × 104 × 2, where 5 represents the number of large grids counted, 104 is the conversion factor from µL to mL and 2 represents the dilution factor of Trypan Blue [12]. Following the experiments, the cells were subjected to trypsinization and counted prior to harvesting their RNA.

### 2.6. Thermotherapy Treatments

Acute and chronic heat treatments were applied to both cell lines. The chronic thermotherapy duration was described by Hooper et al. [11], where the 30 min interval stimulated an exercise bout. The acute thermotherapy was a one-time exposure to 40 °C in a humidified atmosphere containing 5% CO_2_ for 30 min. The chronic thermotherapy constituted a 30 min exposure to 40 °C in a humidified atmosphere containing 5% CO_2_, three times a week, for three weeks. Each cell line was inoculated into 4 wells of a 24-well plate. Upon transferring from a T-75 mL culture vessel, cell count and viability were determined. Viable cells were seeded at a concentration of 10^5^ viable cells/well along with growth medium, totaling a volume of 2 mL. Additionally, 2 mL of growth medium was added into the 4 wells of the plate as a control. The 24-well plate was then incubated at 37 °C in a 5% CO_2_ humidified atmosphere for 48 h. Three 24-well plates were prepared for acute, chronic and control, respectively.

Acute thermotherapy was administered following the 48 h incubation session. Before and after heat exposure, the wells were observed for morphology and confluency. Each plate was subjected to its respective treatments on scheduled days. The chronic thermotherapy protocol was administered following the 48 h incubation session and then incubated at 37 °C in a 5% CO_2_ humidified atmosphere. To serve as a control, no heat treatment, a third plate was seeded and incubated at 37 °C in a 5% CO_2_ humidified atmosphere for the duration of the 3-week thermotherapy treatment. Cells were harvested within 2 h following the last day of each treatment. Following the treatments, the supernatant was collected and cytokine expression was analyzed by an ELISA.

### 2.7. RNA Purification

To isolate high-quality RNA from the samples, Invitrogen TRIzol Reagent (Thermo Fisher Scientific) was used. To the 0.25 mL mixture prepared, 0.75 mL of TRIzol was added. The mixture was homogenized and incubated for 5 min to allow for dissociation of the nucleoproteins complex. To the mixture, 150 µL of chloroform was added and incubated for 3 min. The microcentrifuge tube was centrifuged for 15 min at 12,000× *g* at 4 °C. The aqueous solution was transferred to a new tube.

RNA was precipitated by adding 375 µL of isopropanol, then incubated for 10 min. The mixture was centrifuged for 10 min at 12,000× *g* at 4 °C. The supernatant was discarded and 750 µL of 75% ethanol was added. The tube was briefly vortexed and centrifuged for 5 min at 7500× *g* at 4 °C. The supernatant was removed and the pellet was airdried for 8 min. The RNA was solubilized by being resuspended in 50 µL of RNAse-free water. Both RNA purity and concentration of the extracted samples were quantified using a 1-position spectrophotometer (Thermo Fisher Scientific, Waltham, MA, USA). The samples were stored at −80 °C.

### 2.8. Gene Expression

Gene expression was assessed by performing a real-time polymerase chain reaction (RT-PCR) to qualitatively assess which genes were regulated in each experimental condition. The purified RNA was converted to CT using the TaqMan RNA-to-CT 1-Step Kit (Thermo Fisher Scientific). For a total volume of 20 µL per reaction, 10.0 µL of TaqMan RT-PCR Mix (2×) was mixed with 0.5 µL of TaqMan RT Enzyme Mix (40×) and 9.5 µL of RNA template + RNase-free water. The RNA template recommended was 50 ng per well. The total volume of RNA varied based on the purified concentration of each sample. 

A Custom TaqMan Array 96-well plate (4391524) was formatted with targeted genes found in human skeletal muscles, the assessed genes. Each well of the 96 custom plate was loaded with 20 µL of the sample and a QuantStudio 3 Real-Time PCR Instrument (Thermo Fisher Scientific, Waltham, MA, USA) was programmed based on the manufacturer’s protocol. The experiment was performed and the results were analyzed on Thermo Fisher Cloud using Eukaryotic 18S ribosomal RNA as the endogenous gene and the reference sample being the control of each cell type. Cycle threshold (Ct) values were used to calculate the amount of amplified PCR products relative to the housekeeping gene 18S rRNA and the relative amount of mRNA was calculated as 2^−^^∆∆^^Ct^. Results are expressed as the fold change above the reference sample [13].

### 2.9. Cytokine Inflammatory Markers

Enzyme-linked immunosorbent assays (ELISA) were utilized for the quantification of inflammatory antigens in the cell supernatant. The assessed markers included human Interleukin (IL)-1β, IL-4, IL-6, IL-10 and Tumor necrosis factor α (TNF-α). All ELISA kits were obtained from Thermo Fisher Scientific, with the following kit numbers: BMS224-2 (IL-1β), BMS225-2 (IL-4), EH2IL6 (IL-6), BMS215-2 (IL-10) and BMS223-4 (TNF-α). All reagents were maintained at room temperature before use. Cytokine markers were detected at a wavelength of 450 nm, as per manufacturer’s protocols. A standard curve was used for each assay to determine the cytokine concentration levels of the samples, as per manufacturer’s protocols and expressed in pg/mL.

### 2.10. Statistical Analysis

The data are presented as the mean ± standard error of the mean (Mean ± SEM).

Data collection during the heat treatment was performed in quadruplicate and each sample was analyzed twice with the mean recorded (*n* = 8). The results were analyzed by two-way (time x treatment) analysis of variance (ANOVA) (*p* < 0.05). If sphericity was violated, Greenhouse–Geisser corrections were utilized. Normality was determined using the Shapiro–Wilk test. All group x interactions and main effects are reported as (F_df treatment, df error_) = F statistic, *p*-value and post-hoc analysis were performed using Tukey’s multiple comparisons test at a significance level of *p* < 0.05 to test for significance among the means.

## 3. Results

### 3.1. Skeletal Muscle Cell Viability

**Figure 1:**
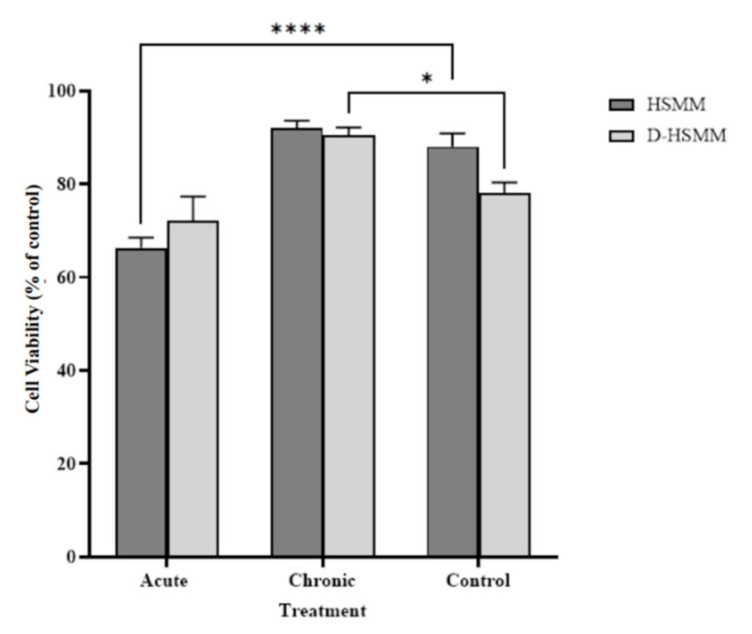
Following the acute and chronic heat treatments, cell viability percentage increased following the chronic treatment but decreased following the acute treatment. The asterisk (*) indicates significant change to the control, where * is *p* < 0.05 and **** is *p* < 0.0001.

Figure 1 shows the change in cell viability of the human skeletal muscle myoblasts following each treatment. Overall, the chronic thermotherapy exposure temperature of 40 °C yielded a slight increase in cell viability, with a significant 1.16-fold increase in D-HSMM, 90.51 ± 1.63, (*p* = 0.01) compared to control; no significant change was noted in the 1.04-fold increase in HSMM following the chronic treatment, 91.93 ± 1.64. The acute treatment caused a significant 1.33-fold decrease in the HSMM sample, 66.3 ± 2.17, (*p* < 0.0001), while the acute exposure on D-HSMM decreased 1.08-fold, but had no significant change in cell viability percentage, 72.15 ± 5.14.

### 3.2. Gene Expression

**Figure 2:**
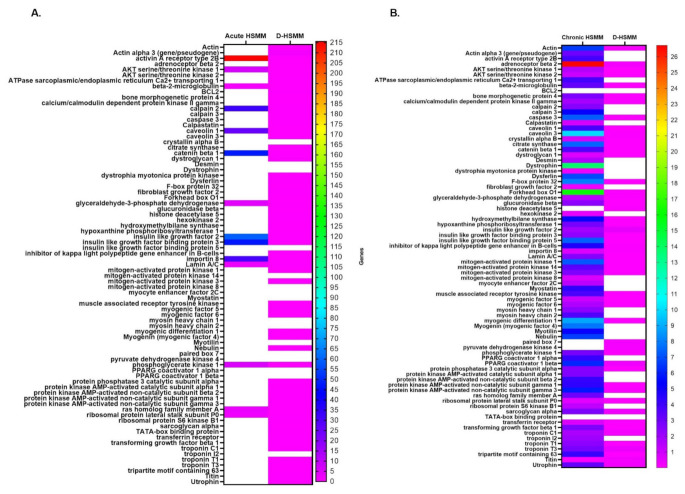
A heatmap of gene expression of the relative qualitative assessed gene in HSMM and D-HSMM following the (**A**) acute and (**B**) chronic heat treatments. All values are expressed in fold-change compared to the control, with eukaryotic 18S rRNA as the endogenous gene.

Figure 2 illustrates the overexpressed genes in HSMM and D-HSMM following the acute and chronic thermotherapies. Each cell type had a control plate serving as the reference plate, while eukaryotic 18S rRNA served as the endogenous gene. Of both cell lines, HSMM yielded a higher value of fold change than D-HSMM. This qualitative assessment shows the fold change as compared to the control. A change in expression was more evident in HSMM following the chronic exposure, while, in D-HSMM, the chronic exposure yielded minimal changes in expression.

HSMM, exposed to the acute treatment, had a range of 1.19–215.61, while the chronic treatment had a range of 0.09–26.71. The D-HSMM exposed to the acute treatment had a range of 0.001–1.64, while the chronic treatment yielded a range of 0.012–0.48. In total, 14 genes were expressed in HSMM following the acute treatment, while 74 genes were expressed following the chronic treatment. In D-HSMM, 40 genes were expressed following the acute treatment, while 51 were expressed following the chronic treatment.

**Figure 3:**
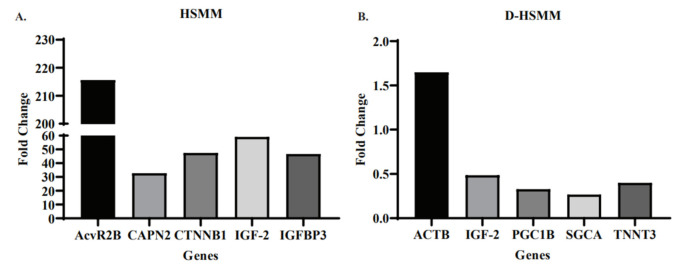
(**A**) The top-5 genes expressed in HSMM following both acute and chronic heat treatments. All five genes expressed the highest change that occurred following the acute treatment, with a range of 32.74–215.61-fold. (**B**) The top-five genes expressed in D-HSMM following both acute and chronic heat treatments. The upregulation of the five genes had a range of 0.26–1.64-fold.

Figure 3 represents the top five genes expressed, regardless of treatment type, in HSMM and D-HSMM. Figure 3A represents the five genes with the highest fold change in the healthy human skeletal muscle myoblasts; the top-five upregulated genes were from the acute treatment. The range was 0.09–215.61-fold. Activin A receptor type 3B (AcvR2B) was upregulated 215.61-fold, followed by insulin-like growth factor 2 (IGF-2), catenin-β1 (CTNNB1), insulin-like growth factor-binding protein 3 (IGFBP3) and calpain 2 (CAPN2), upregulated 59.06-fold, 47.49-fold, 46.71-fold and 32.74-fold, respectively.

Figure 3B represents the five genes with the highest fold change in diabetic human skeletal muscle myoblasts. Excluding actin-β (ACTB), the remaining four genes were upregulated in the chronic treatment; ACTB was upregulated in the acute treatment. The range of upregulated D-HSMM genes was 0.001–1.64, with a mean of 0.07. ACTB was upregulated 1.64-fold, followed by insulin-like growth factor-2 (IGF-2), troponin T3 (TNNT3), PCG coactivator-1β (PGC1B) and sarcoglycan-α (SGCA), upregulated 0.48-fold, 0.39-fold, 0.32-fold and 0.26-fold, respectively.

### 3.3. Cytokine Inflammatory Markers

To determine whether the thermotherapies resulted in cytokine production, cytokines IL-1β, IL-4, IL-6, IL-10 and TNF-α were assessed by ELISA. The soluble cytokines were measured from culture supernatants following a 24 h post-treatment incubation. 

#### 3.3.1. Interleukin-1β

**Figure 4:**
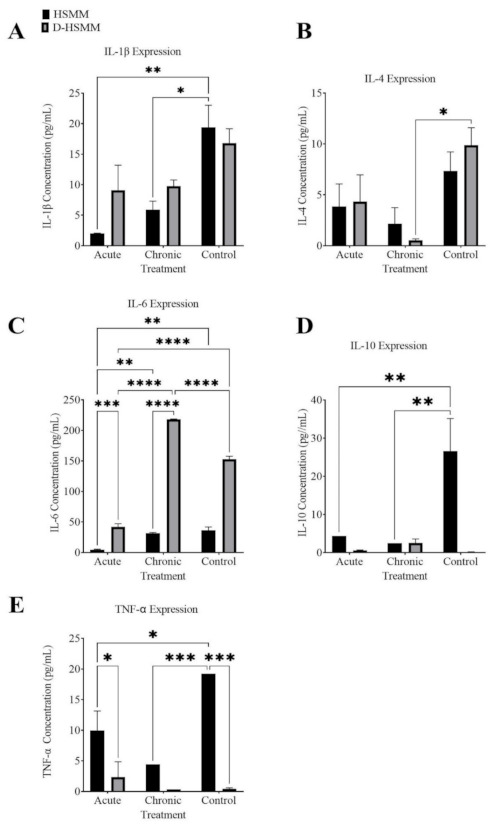
Cytokine concentrations: (**A**) Interleukin-1β; (**B**) Interleukin-4; (**C**) Interleukin-6; (**D**) Interleukin-10; (**E**) Tumor necrosis factor-α, as calculated. The significance was determined when *p* ≤ 0.05 (*), *p* ≤ 0.01 (**), *p* ≤ 0.001 (***), or *p* ≤ 0.0001 (****).

Figure 4A represents the concentration of cytokine IL-1β following both the acute and the chronic treatments on HSMM and D-HSMM cells. A significant effect of the treatments on the results was noted (*p* = 0.0009). A significant decrease was observed in HSMM following the acute treatment (*p* = 0.0036) and following the chronic treatment (*p* = 0.0133), compared to the control. In D-HSMM, a non-significant decrease occurred following the acute (*p* = 0.1388) and the chronic treatment (*p* = 0.1836), compared to the control.

In both HSMM and D-HSMM, the concentrations following the acute and the chronic treatments were lower than those in the control. In HSMM, the acute treatment yielded a 50.43-fold decrease, while the chronic treatment yielded a 2.61-fold decrease. In D-HSMM, the acute treatment resulted in a 1.85-fold decrease, while the chronic treatment had a 1.73-fold decrease.

Analysis of IL-1β concentration showed no interaction between treatment × and cell type (*F*(2,6) = 3.781, *p* = 0.0866). There was an effect of treatment (*F*(2,6) = 27.93, *p* = 0.0009), but not of cell type (*F*(1,6) = 3.670, *p* = 0.1039) on IL-1β concentrations. Tukey’s post-hoc analyses revealed significant differences between acute HSMM and control HSMM (*p* = 0.0036) and between chronic HSMM and control HSMM (*p* = 0.0133). Furthermore, Tukey’s post-hoc analyses also revealed non-significant differences between acute HSMM and chronic HSMM (*p* = 0.6502), acute D-HSMM and chronic D-HSMM (*p* = 0.9998), acute D-HSMM and control D-HSMM (*p* = 0.1388) and between chronic D-HSMM and control D-HSMM (*p* = 0.1836).

#### 3.3.2. Interleukin-4

Figure 4B illustrates the cytokine concentration in HSMM and D-HSMM following acute and chronic heat treatments. A statistical significance was evident in the treatments (*p* = 0.0043). A significant decrease was evident following the chronic treatment on D-HSMM (*p* = 0.0176). No significant change was noted following the acute treatment on D-HSMM (*p* = 0.2507), nor in the acute or chronic treatments on HSMM (*p* = 0.4940 and 0.1891, respectively).

A decrease is apparent following both treatments compared to the control. IL-4 expression in HSMM decreased 1.90-fold following the acute treatment and 3.38-fold following the chronic treatment. The cytokine expression in D-HSMM decreased 2.28-fold following the acute treatment and 18.81-fold following the chronic treatment.

The analysis of the IL-4 concentration showed no interaction between treatment × and cell type (*F*(2,6) = 1.265, *p* = 0.3479). There was an effect of treatment (*F*(2,6) = 15.49, *p* = 0.0043), but not of cell type (*F*(1,6) = 0.1858, *p* = 0.6815) on IL-4 concentrations. Tukey’s post-hoc analyses revealed significant differences between chronic D-HSMM and control D-HSMM (*p* = 0.0176). Furthermore, Tukey’s post-hoc analyses also revealed non-significant differences between acute HSMM and acute D-HSMM (*p* = 0.0176), acute HSMM and chronic HSMM (*p* = 0.9317), acute HSMM and control HSMM (*p* = 0.4940), acute D-HSMM and chronic D-HSMM (*p* = 0.4142), acute D-HSMM and control D-HSMM (*p* = 0.1507) and between chronic HSMM and control HSMM (*p* = 0.1891).

#### 3.3.3. Interleukin-6

Figure 4C represents the concentration of interleukin-6 following both heat treatments. A significant difference was noted in cell type (*p* < 0.0001), treatment (*p* < 0.0001) and the interaction of the two categories (*p* < 0.0001). A significant difference was noted following the acute treatment on D-HSMM and the chronic treatment on D-HSMM (*p* < 0.0001). Compared to the control, the acute treatment had a significant decrease in the cytokine in HSMM (*p* = 0.0012) and D-HSMM cells (*p* < 0.0001). Following the chronic treatment, a significant difference was evident between HSMM and D-HSMM (*p* < 0.0001).

The acute treatment had a significant difference between HSMM and D-HSMM (*p* = 0.0005). Following the chronic treatment, a significant increase was seen in D-HSMM (*p* < 0.0001), compared to the control. In HSMM, the concentration of IL-6 differed between acute and heat treatment (*p* = 0.0030). A non-significant decrease occurred in HSMM following the chronic treatment (*p* = 7670). Following the chronic heat treatment, D-HSMM resulted in a higher expression of IL-6. In HSMM, the expression of IL-6 following both treatments was slightly lower than in the control. The concentration of cytokine expression in HSMM decreased following both treatments but showed an increase in D-HSMM following the chronic treatment (Figure 4C).

The analysis of the IL-6 concentration showed a significant interaction between treatment × and cell type (*F*(2,6) = 391.7, *p* < 0.0001). There was an effect of treatment (*F*(2,6) = 763.6, *p* < 0.0001) and of cell type (*F*(1,6) = 2713, *p* < 0.0001) on IL-6 concentrations. Tukey’s post-hoc analyses revealed significant differences between acute HSMM and acute D-HSMM (*p* = 0.0005), acute HSMM and chronic HSMM (*p* = 0.0030), acute HSMM and control HSMM (*p* = 0.0012), acute D-HSMM and chronic D-HSMM (*p* < 0.0001), acute D-HSMM and control D-HSMM (*p* < 0.0001), chronic HSMM and chronic D-HSMM (*p* < 0.0001 and between chronic D-HSMM and control D-HSMM (*p* < 0.0001). Furthermore, Tukey’s post-hoc analyses also revealed non-significant differences between chronic HSMM and control HSMM (*p* = 0.7670).

#### 3.3.4. Interleukin-10

Figure 4D outlines the cytokine expression of IL-10. Following both treatments, a significant change was evident in the expression of IL-10 based on the interaction (*p* = 0.0036), treatment (*p* = 0.0068) and cell type (*p* = 0.0026). Following the acute treatment, HSMM (*p* = 0.0055) yielded a significant decrease; consequently, IL-10 expression in HSMM also resulted in a significant decrease following the chronic treatment (*p* = 0.0036). Figure 4D also shows an increase in cytokine expression in D-HSMM following both treatments, but a decrease in its expression in HSMM following both treatments. 

The analysis of the IL-10 concentration showed a significant interaction between treatment × and cell type (*F*(2,6) = 16.66, *p* = 0.0036). There was an effect of treatment (*F*(2,6) = 12.85, *p* = 0.0068) and of cell type (*F*(1,6) = 24.57, *p* = 0.0026) on IL-10 concentrations. Tukey’s post-hoc analyses revealed significant difference between acute HSMM and control HSMM (*p* = 0.0055) and between chronic HSMM and control HSMM (*p* = 0.0036). Furthermore, Tukey’s post-hoc analyses also revealed non-significant differences between acute HSMM and acute D-HSMM (*p* = 0.8724), acute HSMM and chronic HSMM (*p* = 0.9912), acute D-HSMM and chronic D-HSMM (*p* = 0.9904), acute D-HSMM and control D-HSMM (*p* >0.9999), chronic HSMM and chronic D-HSMM (*p* > 0.9999) and between chronic D-HSMM and control D-HSMM (*p* = 0.9782).

#### 3.3.5. Tumor Necrosis Factor-α

Following both treatments (Figure 4E), both the acute and the chronic treatment showed a significant change in interaction (*p* = 0.0018), treatment (*p* = 0.0021) and cell type (*p* < 0.0001). Following the acute treatment, a significant change was seen between HSMM and D-HSMM (*p* = 0.0255). Compared to the control, the acute treatment on HSMM also resulted in a significant change (*p* = 0.0103). A significant decrease was evident following the chronic treatment on HSMM (*p* = 0.0008), compared to the control. 

As outlined in Figure 4E, both acute and chronic treatments resulted in a decrease in TNF-α expression in HSMM. However, in D-HSMM, the acute treatment resulted in an upregulation of the cytokine, while the acute treatment resulted in a decrease in its expression. 

The analysis of the TNF-α concentration showed a significant interaction between treatment × and cell type (*F*(2,6) = 21.62, *p* = 0.0018). There was an effect of treatment (*F*(2,6) = 20.50, *p* = 0.0021) and of cell type (*F*(1,6) = 114.1, *p* < 0.0001) on TNF-α concentrations. Tukey’s pos-hoc analyses revealed significant difference between acute HSMM and acute D-HSMM (*p* = 0.0255), acute HSMM and control HSMM (*p* = 0.0103), chronic HSMM and control HSMM (*p* = 0.0008) and between chronic D-HSMM and control D-HSMM (*p* > 0.9999). Furthermore, Tukey’s post-hoc analyses also revealed non-significant differences between acute HSMM and chronic HSMM (*p* = 0.0958), acute D-HSMM and chronic D-HSMM (*p* = 0.8147), acute D-HSMM and control D-HSMM (*p* = 0.8458) and between chronic HSMM and chronic D-HSMM (*p* = 0.2632).

## 4. Discussion

**Figure 5:**
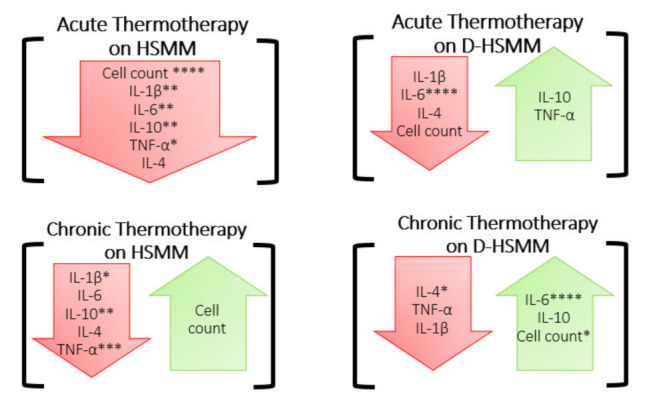
Summary of the findings of this study. The red arrows indicate a decrease, compared to the control, while the green arrows indicate an increase, compared to the control, following each heat treatment. The significance was determined when *p* ≤ 0.05 (*), *p* ≤ 0.01 (**), *p* ≤ 0.001 (***), or *p* ≤ 0.0001 (****).

Cell-density percentage was investigated by exposing both muscle cell types (HSMM and D-HSMM) to acute and chronic heat treatments. The goal was to determine whether the cell viability remained constant, compared to the control, when exposed to a 40 °C environmental temperature. The data indicate an acute exposure to an elevated temperature, 40 °C, on D-HSMM, significantly decreases cell-density percentage. In contrast, chronic heat exposure, on both HSMM and D-HSMM, yields a higher cell-density percentage (Figure 1 and Figure 5). The lower density percentage expressed in D-HSMM, compared to HSMM, was expected; comparing Certificate of Analysis, provided by the supplier, of the cell lines, D-HSMM underwent a longer doubling time than HSMM—49 h and 19 h, respectively.

Previous studies reported distress caused by Type 2 diabetes on muscle metabolism [12]. Outcomes of the disease on skeletal muscle include an aberrant lipid deposition and a decrease in intermyofibrillar mitochondrial content, thus impeding the cell response to insulin through its ability to alternate between fat and carbohydrate oxidation [14,15,16]. This may have influenced cell viability, wherein, in this study, the cell-density percentage of D-HSMM was lower than HSMM in all treatment conditions.

Several studies using cultured muscle cells and animal models demonstrated that heat stress increases muscle protein synthesis [17,18,19], along with skeletal muscle mass [20]. In this study, the chronic heat exposure resulted in a higher density percentage of cell viability; HSMM was increased 1.04-fold, while D-HSMM increased 1.16-fold. In a human model, heat stress-associated muscle hypertrophy increased muscle strength in adult men [21,22]. It has been suggested that heat stress induces muscle hypertrophy by activating intracellular pathways, including phosphatidylinositol 3-kinase-Akt and the extracellular signal-regulated kinase [23]. This pathway inhibits the upregulation of dexamethasone-induced upregulation of the atrophy-induced ubiquitin ligases by obstructing the nuclear translocation of the FOXO transcription factor [24]. The extracellular signal-regulated kinase induces gene expression in response to hypertrophic stimuli [20], referred to as a mitogen-activated protein kinase.

An array of genes was qualitatively analyzed following each treatment. For the healthy human muscle myoblasts cell line, the top-five genes expressed with the highest fold-change occurred after exposure to the acute treatment. However, in the diabetic human muscle myoblast cell line, the top-five genes expressed with the highest fold-change, compared to the control, occurred mostly following the chronic treatment, except for Activin A receptor type 2B. Activin A receptor type 2B (AcvR2B) was upregulated in HSMM and hindered in D-HSMM—215.613 and 0.001, respectively. AcvR2B ligands myostatin and activins inhibit muscle hypertrophy [25,26]. Activins negatively affect regulators of muscle mass through their regulatory role of TFG-β [26]. An upregulation of this gene following the acute treatment on HSMM suggests the possibility of muscle wasting, which was reflected in the observed decrease of cell viability percentage following the acute treatment.

Insulin-like growth factor 2 (IGF-2) provides instructions to the cell on making IGF-2 protein, promoting cell growth and proliferation [27]. The observed upregulation of IGF-2 suggests cell growth and proliferation in D-HSMM (Figure 2B), following the chronic treatment, and in HSMM, following an acute treatment (Figure 2A).

While Catenin-β-1 (CTNNB1) is a transcriptional activator, it also promotes myogenesis and growth of various tissues [28]. In cellular growth, β-catenin is identified through its recognized role as a transcription factor [29] and induction of Myc proto-oncogene protein (c-Myc) and cyclin D1 [28]. This induction can occur directly or indirectly through the paired-like homeodomain transcription factor2 [28]. This indirect induction is evident in skeletal muscles exposed to in vitro stimulation of Wnt/β-catenin [30]. Cyclin D1 and c-Myc are both critical transcription factors in cell development, proliferation and apoptosis. In this study, while both HSMM and D-HSMM expressed an upregulation of catenin-β-1, the HSMM cell line expressed it at a higher fold. Additionally, the acute bout of 40 °C resulted in a higher expression of catenin-β-1 on HSMM, which could be explain by the fact that a one-time exposure is a sudden change to the cell environment, whereas, in the chronic treatment, the cells may potentially acclimate to the changes over three weeks.

Insulin-like growth factor-binding protein 3 (IGFBP-3) plays a critical role in the physiological function of insulin growth factor 1 (IGF-1) [31]. The expressed gene is known as the main circulating carrier of IGF-1 and the high expression in this study indicates the over-activation of the IGF-1 protein. Calpain-2 (CAPN2) is part of the calcium-activated protease, which plays a role in exocytosis, cell fusion, apoptosis and proliferation [32,33]. The upregulation of CAPN2 correlates with a decrease in cell density percentage in HSMM following the acute thermotherapy, thus suggesting CAPN2 acts as an apoptotic agent following a sudden elevation in environmental temperature.

Actin-β codes for β-actin proteins that assist in forming the structural framework inside cells [34] and the elevation, evident following the acute treatment (Figure 2), suggests an increase in actin activity [34] and cellular mobility responses [35,36]. Over-expression is necessary for cellular responses requiring constant and stable concentration levels [35], which was reflected in D-HSMM following the acute treatment, indicating that the one-time exposure promoted the cells to upregulate β-actin as a response to the abrupt change in environmental temperature.

A qualitative increase in troponin T3 (TNNT3) indicates an upregulation in fast skeletal troponin T protein, part of the fast-twitch skeletal muscle [37]. Additionally, TNNT3 positively regulates the expression of Ca^2+^ channel α1 (Cav1.1) [38], where a decrease in Cav1.1 expression is associated with skeletal muscle loss [39]. In this study, the upregulation of TNTT3 in D-HSMM (0.399-fold) and HSMM (2.808-fold) following the chronic treatment may suggest an increase in the amount of Cav1.1, resulting in improved skeletal function, as reflected in the observed increase in cell viability following the chronic treatment. Moreover, this may also present a new therapeutic approach for the diabetic population, where chronic thermotherapy upregulates TNTT3 to diminish the deleterious effects of aging and disease on muscle function. 

The Peroxisome Proliferator-Activated Receptor-gamma coactivator-1β (PCG-1β) are positive regulators of skeletal muscle mass and energy metabolism [40]. An upregulation following the chronic thermotherapy on D-HSMM suggests an increase in protein synthesis and myotube diameter. The sarcoglycan-α (SGCA) encodes a portion of the dystrophin–glycoprotein complex (DGC), which is vital in the stability of muscle fiber membranes and the linking of the actin cytoskeleton [41]. An elevation in this gene in the D-HSMM cell line suggests that chronic thermotherapy upregulates members of the DGC to protect the sarcolemma from mechanical stress in an attempt to maintain membrane integrity. 

Interleukin-1β and TNF-α are pro-inflammatory mediators usually referred to as external stress signals that function as organismal stress or injury indicators in other cells [42]. IL-1β is released in response to inflammatory or infectious stimuli [43]. In previous studies, IL-1β was reported to be upregulated in an acute setting and IL-1β was involved in the metabolic adaptation of skeletal muscle tissues as a response to non-infectious stress [43]. In this study, the concentration of IL-1β showed a significant decrease in the cytokine expression in HSMM following the acute treatment (*p* = 0.0036) and following the chronic treatment (*p* = 0.0133). The cytokine expression in D-HSMM also had no significant change following the acute treatment (*p* = 0.1388) or the chronic treatment (*p* = 0.1836). Overall, the treatments yielded a significant change in cytokine expression (*p* =0.0009). The concentration of IL-1β decreased after the treatments, compared to the controls. In HSMM, the cytokine expression decreased 9.75-fold following the acute treatment and 3.27-fold following the chronic treatment. In D-HSMM, a 1.85-fold decrease was noted following the acute treatment, while a 1.73-fold reduction was noted following the chronic treatment (Figure 4 and Figure 5). The observed decrease in IL-1β expression correlates with recent studies that report that a decrease in the expression of pro-inflammatory markers is associated with heat stress. These data also suggest thermotherapy may increase the levels of heat shock factor (HSF)-1 and suppress pro-inflammatory markers through competitive inhibition on NF-κβ binding [44].

Overexpression of TNF-α and IL-10, following an injury, is expected and indicates normal muscle repair [45]. However, following hyperthermia exposure, circulating TNF-α is either delayed, lessened or completely absent [45,46,47,48,49]. In this experiment, both the acute and chronic experiments (*p* = 0.0022) resulted in a significant change in TNF-α expression in both cell lines (*p* < 0.0001): a 1.93-fold and 4.36-fold decrease in expression in HSMM following the acute and chronic treatments, a 1.36-fold decrease in D-HSMM following the chronic experiment and a 5.26-fold increase in D-HSMM following the acute heat treatment. Following the acute and chronic heat treatments, the expression of TNF-α in HSMM significantly decreased (acute, *p* = 0.103; chronic, *p* = 0.0008). While a decrease was also evident following the chronic heat treatment on D-HSMM, the change was not significant. However, the acute treatment (*p* = 0.0254) showed a significant change between cell types—a 1.92-fold decrease in HSMM and a 5.26-fold increase in D-HSMM (Figure 4 and Figure 5). Generally, TNF-α is a pro-inflammatory marker involved with the signaling of apoptosis and necrosis [50], through binding to the TNF receptor (TNFR)1 [51], which utilizes signal transduction to regulate apoptosis [51]. A down-regulation of TNF-α suggests an active cytoprotective role of HSP70 [52] by recruiting HSF-1 transcription factors intracellularly and extracellularly. Our data suggest acute or chronic thermotherapy may decrease pro-inflammatory markers in skeletal muscles, thus protecting cellular homeostasis. 

Interleukin-4, a pleiotropic anti-inflammatory cytokine [53], is known to play various roles in homeostatic regulation and disease pathogenesis [54]. While previous studies reported an upregulation of IL-4 following strength training [53], in this study, the concentration of IL-4 was significantly reduced following both acute and chronic treatments (*p* = 0.0043). A decrease in cytokine expression in HSMM was noted following the acute (1.90-fold) and the chronic (3.38-fold) treatments. A decrease was also evident in D-HSMM following the acute (2.28-fold) and chronic (18.81-fold) treatments. Additionally, the decrease in D-HSMM following the chronic treatment was significant (Figure 4 and Figure 5). This cytokine regulates myoblast fusion with myotubes, thus promoting muscle cell fusion and growth [55] when muscles are damaged by exercise. While there was a decrease in the cytokine expression, the acute treatment resulted in a higher concentration of IL-4 than the chronic treatment. The observed decrease in IL-4 expression may be associated with the deactivation of the humoral immune system linked to acute or chronic heat stress.

Skeletal muscle produces interleukin-6 in various conditions, including local muscle injury, inflammation, exercise and hypoperfusion [56]. This pleiotropic cytokine has both anti-inflammatory and pro-inflammatory properties [57]. The production is usually a response to receptor-mediated signals and disturbances in the internal cell hemostasis [9]. Interleukin-6 attaches to its receptor through the classic or trans-signaling pathway, which is negatively regulated through suppressor cytokines, including Janus kinase (JAK) and signal transducer and activator of transcription (STAT) molecules [58]. In events of inflammation, IL-6 acts as an anti-inflammatory cytokine by directly acting on plasma cells and indirectly promoting Bcl6-dependent follicular CD4 T cell differentiation, thus promoting antibody production [58]. At temperatures above 40.5 °C, in humans, the translational arrest of nonessential proteins, non-HSPs, is due to the inhibition of ribosomal initiation factors [9]. Since skeletal muscle cells are capable of synthesizing and secreting IL-6 during hyperthermia, one study indicates this as IL-6 heat-shock resistance in skeletal muscles [9]. Through its role as an anti-inflammatory marker, IL-6 can suppress inflammatory responses by reducing the expression of pro-inflammatory cytokines TNF-α and IL-1β. Consequently, in hyperthermia, circulating levels of IL-6 and IL-10 are upregulated, while levels of TNF-α and IL-1β are absent [47]. This was reflected in D-HSMM following the chronic treatment, where IL-6 expression was upregulated 1.43-fold.

One explanation for IL-6 being significantly upregulated compared to the remaining assessed interleukins is the selective production of HSP, which could preserve the availability of other proteins involved in regulation, as evident in HSP72 inhibiting NF-κβ signaling, altering IL-6 regulation in LPS-induced cells [59]. The observed decrease in concentrations of IL-6 indicates a plausibility in the cell lines being sensitive to insulin, which was more evident in the acute treatment group. However, the chronic treatment demonstrated an upregulation of IL-6 (*p* < 0.0001) (Figure 4 and Figure 5). In relation to these findings, Welc et al. (2012) reported an upregulation of the IL-6 gene expression during hyperthermia in a mouse cell culture. Following acute exposure to 42 °C, the expression of IL-6 was upregulated, leading to an inhibition of TNF-α mRNA [46]. The observed increase of IL-6 in the diabetic cells subjected to the chronic heat treatment may indicate its anti-inflammatory effect by promoting an increase in cellular viability by reducing inflammation, which was also observed in the downregulation of pro-inflammatory markers TNF-α and IL-1β.

Both IL-6 and IL-10 are regulated by HSF-1, where previous studies found that the inhibition of HSF-1 yielded an upregulation in IL-6 [45]. Interleukin-10 is an anti-inflammatory cytokine, that, when subjected to heat shock, can yield an early response and provide components for transcriptional messages to induce an early stress-induced immune response [45]. In this study, the expression of IL-10 showed a significant change following the acute and chronic treatments (*p* = 0.0068) in both the HSMM and D-HSMM cell lines (*p* = 0.0026). IL-10 expression was upregulated in D-HSMM following both heat treatments but was down-regulated in HSMM following the treatments. While the change was not significant, the chronic treatment resulted in an upregulation of the cytokine in D-HSMM and a significant down-regulation in HSMM (*p* = 0.0036); additionally, the acute treatment resulted in a significant decrease in the cytokine expression in HSMM (*p* = 0.0055) (Figure 4 and Figure 5). 

In D-HSMM, the cytokine was overexpressed 3.59-fold following the acute treatment and 15.99-fold following the chronic treatment. In HSMM, the cytokine expression decreased 6.09-fold following the acute treatment and 10.89-fold following the chronic treatment. This was not expected, since IL-10 was over-expressed in previous studies [46,47]. The expression of IL-10 decreased in both HSMM and D-HSMM when exposed to the heat treatments, except in D-HSMM, where a non-significant increase occurred following the chronic treatment. Such a decrease in the expression of IL-10 may be linked to the low concentrations of pro-inflammatory marker TNF-α. Similar to the report by Park et al. (2007), TNF-α positively correlates to the expression of IL-10 by macrophages [60]. The observed increase in the concentration of IL-10 following the chronic treatment on the D-HSMM cell line may benefit the cell by preventing the upregulation of phagocytic and dendritic cells.

The difference in the patient’s age and BMI may be a limitation to the findings of this study. The reported increase in several genes due to heat treatment was based on a singular and exploratory analysis. We did not establish whether the myocytes differentiated into myotubes at confluency, as differentiation can occur spontaneously, although to a limited extent. Overall, our study only gives preliminary and exploratory information about the changes in gene expression associated with thermotherapy in type 2 diabetic and normal, healthy non-diabetic skeletal muscle cells.

## 5. Conclusions

The data reported help outline inflammatory and gene regulation changes following thermotherapy applied to skeletal muscle cells. The heat exposure induced changes favoring an anti-inflammatory pathway with a decrease in the expression of IL-1β and TNF-α. The pattern of cytokine expression showed the anti-inflammatory effects of IL-6 regarding the suppression of IL-1β and TNF-α. Chronic or acute thermotherapy did not significantly affect the concentration of circulating cytokines IL-1β and IL-10. In this study, IL-10 expression in D-HSMM was increased following both the acute and chronic treatments, which highlights its role as an anti-inflammatory cytokine. TNF-α expression was also decreased, except for D-HSMM exposed to the acute treatment, indicating TNF-α was suppressed by various factors, including IL-6. Chronic thermotherapy could be beneficial for the diabetic population where anti-inflammatory markers are upregulated to maintain cellular homeostasis and pro-inflammatory markers are down-regulated. The gene expression array plate provided a qualitative analysis of various biomarkers applicable to the skeletal muscle cell line. The results demonstrate skeletal muscles exhibit a different pattern of gene expression when exposed to acute treatment or chronic treatment. These findings allude to chronic thermotherapy as a promising and inexpensive tool for sedentary patients with Type 2 Diabetes by improving adverse inflammatory effects associated with diabetic skeletal muscle cells. Additional studies are needed to evaluate the role of HSP70 in relation to heat treatment.
